# Domestic violence management in Malaysia: A survey on the primary health care providers

**DOI:** 10.1186/1447-056X-7-2

**Published:** 2008-09-29

**Authors:** Sajaratulnisah Othman, Noor Azmi Mat Adenan

**Affiliations:** 1 School of Primary Health Care, Monash University, Australia; 2 Department of Primary Care Medicine, Faculty of Medicine, University of Malaya, Malaysia; 3 Department of Obstetric & Gynaecology, Faculty of Medicine, University of Malaya, Malaysia

## Abstract

**Aim:**

To assess the knowledge, attitudes and practices of primary health care providers regarding the identification and management of domestic violence in a hospital based primary health care setting.

**Method:**

A survey of all clinicians and nursing staff of the outpatient, casualty and antenatal clinics in University Malaya Medical Centre using a self-administered questionnaire.

**Results:**

Hundred and eight out of 188 available staff participated. Sixty-two percent of the clinicians and 66.9% of the nursing staff perceived the prevalence of domestic violence within their patients to be very rare or rare. Majority of the clinicians (68.9%) reported asking their patients regarding domestic violence 'at times' but 26.2% had never asked at all. Time factor, concern about offending the patient and unsure of how to ask were reported as barriers in asking for domestic violence by 66%, 52.5% and 32.8% of the clinicians respectively. Clinicians have different practices and levels of confidence within the management of domestic violence. Victim-blaming attitude exists in 28% of the clinicians and 51.1% of the nursing staff. Less than a third of the participants reported knowing of any written protocol for domestic violence management. Only 20% of the clinicians and 6.8% of the nursing staff had ever attended any educational program related to domestic violence.

**Conclusion:**

Lack of positive attitude and positive practices among the staff towards domestic violence identification and management might be related to inadequate knowledge and inappropriate personal values regarding domestic violence.

## Introduction

Domestic violence is a major social and medical problem. It occurs in all countries irrespective of social, economic, cultural or religious values. The World Health Organization (WHO) has reported that population studies around the world have found 10 to 69% of women reported being physically assaulted by an intimate partner at some point in their lives [1]. In Malaysia, 39% of women above 15 years of age were estimated to have been physically beaten by their partner [2]. A study in the outpatient clinic of the University Malaya Medical Centre (UMMC) revealed that one in seven female patients attending the clinic had a background of domestic violence [3].

The impact of domestic violence is alarming. Fatalities are related to partner homicides or women committing suicide [1,4,5]. Morbidity as the consequences of domestic violence comes in the form of poor health status, poor quality of life and high use of health services [6,7]. Many abused women suffer acute physical injuries and many other chronic health problems that present as ambiguous symptoms and physical findings [8,9]. Psychosomatic complains and non-specific chronic pains are common [9]. These presentations may be treated in health care facilities without identification of the underlying cause, leaving the patient at risk for subsequent episodes of abuse [10-14].

Abuse usually escalates during pregnancy and represents a significant risk to the health of both mother and infant [15]. Abused pregnant women have been reported to have late and poor antenatal check-up when compared to pregnant women without an abusive background [16]. The impacts of domestic violence also extend to children of the abused women. A survey by Women Safety Australia (1996) found that 60% of the abused women have children under their care during the abuse and 38% of them reported that their children witness the violence episodes [17]. These children suffered injury when they were caught between their fighting parents [18]. Children brought up in a home where domestic violence occurs have the tendency to develop behavioral or psychological problems, with risk of poor health in later life [19]. There is a close association between domestic violence and child abuse and it is estimated that child abuse occurs in 50% of families with domestic violence [17].

Studies have shown that most of the abused women will keep their experiences to themselves [20-22]. Those who sought help were most likely to disclose their experience to their close relatives or friends for help [17]. In view of their poor health, these abused women were noted to be frequent attendees to health care facilities [15]. However, only a small proportion of these victims were successfully identified. It was noted that the lifetime disclosure rate for abuse women to be around 30% and a low general practitioners' inquiry rate of 13% despite high levels of prevalence among those attending health care facilities [23,24].

Factors such as shame, embarrassment, fear of partner's retaliation and perception that it is not the doctor's role to intervene are factors that prevent abused women from seeking help from health care providers [12,25]. Conversely, primary issues like lack of time, inadequate training, uncertainty about how to respond and perceptions of patient non-compliance affect professional response to domestic violence from these doctors [26-28].

The medical practitioner's personal value system and beliefs about domestic violence also play an important role. A study in an emergency department in Hong Kong reported that the doctors found it difficult to optimally manage victims of domestic violence because of the belief in the importance of maintaining family unity and that domestic violence is a private issue [29]. Fewer doctors were found to screen for domestic violence compared with other behavioral risks, such as alcohol and drug consumption, and risk of HIV/AIDS [30]. More doctors also believed that domestic violence intervention was less successful than intervention for tobacco and HIV/AIDS risks [30].

The aim of this study was to assess the knowledge, attitude and practices of primary health care (PHC) provider teams (clinicians and nursing staff) related to the identification and management of domestic violence. The information gathered hopefully may assist in the development of appropriate intervention strategies that lead to improvements in the management of domestic violence.

## Method

### Setting and study participants

A cross-sectional study was carried out at three PHC clinics of University Malaya Medical Centre (UMMC) from October to December of 2002. The patients make their first contact with health care providers at these PHC clinics. These clinics are managed by the Primary Care Medicine/Outpatient department (PMC), the Accident and Emergency department (A&E), and the Obstetric and Gynecology department (O&G).

All health care providers who were working at any of the three clinics during the survey time were invited to participate. They consisted of the clinicians (consultants, specialists and medical officers (MOs) who were either master students or servicing doctors) and nursing staff (sisters, staff nurses and assistant nurses and medical assistants).

### Questionnaire

The survey instrument was a questionnaire, adapted from a study by Sugg et al (1999), that seek to find different responses from the participants on various aspects of domestic violence management [31]. Six different main categories of responses were assessed by the questionnaire:

• Frequency of domestic violence screening

• Provider self-efficacy

• Safety concerns

• Blaming the abused person

• Concern of offending the patients

• Perceived system support.

Questions on screening frequency and provider self-efficacy using a 5-point Likert scale, ranging from strongly negative to strongly positive to categorize the responses. The questionnaires for the nursing staff have a slight variation from those to the clinicians. Questions specific to clinician's medical consultation that were outside the boundary of nursing tasks were omitted for the nursing staff. This study was initially piloted for its suitability with the community studied before it was fully conducted.

### Procedure

Ethics approval for the study was granted by the Medical Ethics Committee of UMMC. After distribution of the questionnaires, the participants were given time to complete the questionnaire and to return it via internal mail. Written informed consent was taken from the participants. The participants were not asked of their name in order to ensure anonymity. However, all the questionnaires were individually numbered to allow tracking of non-responses. To ensure confidentiality of the participants, a research assistant carrying out the tracking was the only one who had the access to the list of individuals. The non-responders were followed-up with three successive phone calls.

### Data Analysis

The questionnaire responses were coded and the frequencies were tabulated. The participants were divided into two groups based on their job description: the 'clinician group' and the 'nursing group' Data analyses were carried out separately on each group. The χ^2 ^tests of goodness of fit were used to determine whether the distributions of responses to the specific questions departed significantly from chance. The result is significant with *p *< 0.05. Type I error rate was set at 0.05 for each test.

## Results

Out of 188 staff available at the time of the study, only 108 responded to the questionnaires. The clinicians were made up of 61 respondents with response rate of 72.6%. They consisted of 18 consultants/physicians and 43 MOs. The rest of the respondents were from the nursing staff and consisted of 42 nurses and five medical assistants. The low response rate (45%) of the nursing staff has been contributed by difficulty in making contact with the participants in view of shift working time and perhaps minimal research culture exposure among them. Sixty-eight percent of the participants were female and nearly all (98.7%) of the participants had been in service for five years or more. Three of the clinicians (5%) and two of the nursing staff (4%) had personal experience of domestic violence at some point in their lives.

### Perceived prevalence of the problem

Table 1 shows that majority of the respondents (62.3% of the clinicians and 65.9% of the nursing staff) perceived that domestic violence within the patients attending their clinic was either very rare or rare.

**Table 1:** Perceived prevalence of domestic violence among patients †

	Perceived prevalence of domestic violence among patients‡			
	
	Very rare	Rare	Common	Very common
Clinicians (n = 61)	13.1	49.2	36.1	1.6
Nursing staff (n = 47)	17	48.9	31.9	2.1

† Clinicians and nursing staff were asked the question, 'What do you think of the prevalence of domestic violence among patients attending your clinic?'

‡ Observed values are given as percentage of clinicians and nursing staffs

As shown in Table 2, 77% of the clinicians and 63.6% of the nursing staff had identified at least once a patient with domestic violence experience in their work. Of these health care providers, 64% of the clinicians and 71.4% had identified at least one patient who had been abused in the past year. Only 32.8% of the clinicians and 15.6% of the nursing staff had ever identified a perpetrator/abuser.

**Table 2:** Identification of abused victims or perpetrators/abusers

	Identification of abused victims or perpetrators/abusers †‡	
	
	Clinicians (n = 61)	Nursing staff (n = 47)
Ever identified an abused victim	77	63.6
Have identified at least an abused victim the past one year	64	71.4
Have ever identified a perpetrator/abuser	32.8	15.6

† Observed values are given as percentage of clinicians and nursing staff

‡ Missing values are not included

Most of the respondents (86.7% of the clinicians and 73.9% of the nursing staff) believed that they had a role to play in the management of domestic violence.

### Self-reported asking about abuse

While the majority of clinicians (68.9%) reported asking about abuse to their patients at 'times', 26.2% had never screened any of their patients for domestic violence.

### Frequency of asking about domestic violence

The frequency of clinicians in asking about domestic violence when seeing various clinical presentations is shown in Table 3. When seeing someone with injuries, 52.5% of clinicians almost always/always ask about abuse. Patients with chronic pelvic pain, headache, irritable bowel syndrome, unexplained intrauterine growth retardation (IUGR) and those lack prenatal care were seldom/never asked regarding abuse by more than 50% of clinicians. With the exception of the question on depression or anxiety, all other observed distributions of responses differed significantly from chance.

**Table 3:** Frequency of clinicians asking about abuse (n = 61) †

	Frequency of clinicians asking about abuse ‡				
			
	Almost always or Always	Sometimes	Seldom or Never	Statistical test value χ^2^	*p *value
Injury	52.5	29.5	18.0	11.25	0.005 <*p *< 0.0005
Depression or anxiety	41.0	26.2	32.8	1.99	NS
Chronic pelvic pain	6.6	31.1	62.3	28.56	0.005 <*p *< 0.0005
Headache	8.2	34.4	57.4	22.16	0.005 <*p *< 0.0005
Irritable bowel syndrome	3.3	19.7	77	54.9	0.005 <*p *< 0.0005
Unexplained IUGR	6.6	16.4	77	53.35	0.005 <*p *< 0.0005
Premature labor	8.2	14.8	77	52.86	0.005 <*p *< 0.0005
Lack prenatal care	16.4	31.1	52.5	12	0.005 <*p *< 0.0005

† Clinicians were asked the question 'How frequent do you asked about domestic violence when seeing a patient with these conditions?'

‡ Observed values are given as percentage of clinicians. All expected percentages are 33.33%.

### Barriers to asking violence

Barriers to asking about domestic violence have been reported to be lack of time (65.6% of clinicians), afraid of offending their patients (52.5% of the clinicians) and unsure of how to ask (32.8% of the clinicians). Of those clinicians who reported being afraid of offending their patients, 71.4% were O&G clinicians, 62.5% were A&E clinicians and 28% were outpatient clinicians. The concern of offending the patients was only present in 30% of the nursing staff.

### Confidence in asking

Clinicians' confidence in asking about various health issues is shown in Table 4. A high proportion of clinicians reported being very confident when asking about smoking and alcohol compared to asking other health presentations. Overall, clinicians felt more confidence in asking about high risk behaviors than about different types of abuse. There was no significant departure from chance in the distribution of responses on physical and sexual abuse.

**Table 4:** Confidence of clinicians in asking about various health presentations (n = 57) †

	Confidence levels of clinicians ‡§				
			
	Not at all or Slightly	Moderately	Very confident	Statistical test value χ^2^	*p *value
Smoking	3.5	12.3	84.2	67.0	0.005 <*p *< 0.0005
Alcohol	5.3	21.1	73.7	43.9	0.005 <*p *< 0.0005
Sexual behavior	17.5	49.1	33.3	8.5	0.025 > *p *> 0.01
Emotional abuse	40.4	43.9	15.8	8.0	0.025 > *p *> 0.01
Physical abuse	24.6	42.1	33.3	2.6	NS
Sexual abuse	42.1	38.6	19.3	5.16	NS

† Clinicians were asked 'How confident are you in asking the following?'

‡ Missing values are not included.

§ Observed values are given as percentage of clinicians. All expected percentages are 33.33%.

### Concern about safety

A very small percentage of participants (6.6% of clinicians and 17.8% of nursing staff) expressed concern about their own safety when asking about domestic violence. However, 41% of the clinicians and 13% of the nursing staff expressed concern that it may increase the risk of further abuse of patients with a background of domestic violence.

### Blaming the abused person

Twenty-eight percent of the clinicians and 51.1% of the nursing staff endorsed the item stating that 'the abuse person usually has done something that would trigger the perpetrator to abuse them'.

### Making police report

Of all the participants who answered the question 'Would you make a police report of domestic violence even if your patient objects to it?' 32.8% of the clinicians and 24.4% of the nursing staff answered 'yes'.

### Perceived self-efficacy

Providers' self-efficacy in managing domestic violence is shown in Table 5. Most clinicians (53.4%) reported having minimal or no strategies with only 45% of them reported having moderate efficacy to help abused women. Eighty percent of clinicians reported having few or no strategies to help the abusers. Very few clinicians perceived themselves as having effective strategies in the management of domestic violence. In all cases, the observed distributions differed significantly from the distributions expected by chance alone.

**Table 5:** Perceived self-efficiency of clinicians in management of domestic violence (n = 60) †

	Perceived self-efficiency of clinicians ‡§				
			
	Not at all or Slightly	Moderately	Very efficient	Statistical test value χ^2^	*p *value
Strategies to help abused patients	53.4	45	1.7	27.7	0.005 <*p *< 0.0005
Strategies to help the abusers	80	20	0	62.4	0.005 <*p *< 0.0005
Confidence in referring abused patients	31.7	56.7	11.7	18.3	0.005 <*p *< 0.0005
Access to different management information	54.2	40.7	5.1	22.45	0.005 <*p *< 0.0005

† Clinicians were asked 'How efficient are you in different management of domestic violence'

‡ Missing values are not included

§ Observed values are given as percentage of clinicians. All expected percentages are 33.33%.

### Perceived system support

#### Aware of any written protocol

Less than a third of the participants (28.7%) reported knowing of any written protocol for the management of domestic violence victims.

#### Social worker

Ninety-three percent of the participants believe that having a social worker could support them in the management of domestic violence victims. However, only 72.1% reported having easy access to social worker. Around 10% of the participants were unsure of the availability of social worker at their work place.

#### NGO

Less than half of the participants knew of any non-governmental organizations that help support domestic violence victims.

#### Previous education on domestic violence

Only 20% of the clinicians and 6.8% of the nursing staff had ever attended any educational program. Of these, one third of the clinicians and none of the nursing staff had attended a program in the past one year.

## Discussion

This study yielded important information about the current perceptions and approaches of the health care providers in Malaysia towards the identification and management of domestic violence in the primary care setting. The findings will be useful in guiding the development of appropriate clinical interventions to improve care in primary care settings in general as well as specifically in the UMMC.

However, caution should be taken when interpreting the results of this study in view of several limitations of this study. First, this study was a single study which was carried out in a single primary care location. Thus, the findings may not be applicable to other medical settings, such as in the inpatient or mental health settings. Second, the UMMC is a large public tertiary teaching hospital with its location in a suburban setting and because of this, the findings may not be representative of smaller primary care settings such as the general practice clinics or other medical facilities from non-urban locations. Third, since the data collection was based on self-reporting by the participants, respond and recall bias may result in desirable answer despite the confidential manner of the data collection.

As evident in this study, more than sixty percent of health care providers in UMMC believed that the prevalence of domestic violence among patients attending their clinic to be low. This may be one of the many causes of low screening for domestic violence cases. Seventy percents of clinicians have reported screening for their patients but at times only. There was only one doctor among the 61 clinicians who screened all his/her patients for domestic violence.

Despite perception of low prevalence in domestic violence cases, 65% of the clinicians identified an abused victim within the past year. This is contrary to physicians' perception of low prevalence of domestic violence when the actual prevalence of domestic violence was found to be higher in those attending the health care facilities when compared to population [32]. The prevalence of domestic violence cases among patients in various primary care settings varies from 8.5% to 41% [6,32-36].

Physical injuries related to abuse may be one of the most obvious symptoms presented to medical facilities. However, only half of the clinicians reported 'always or almost always' asking their patients for any underlying abuse when treating cases of injury. This reported practice of inquiring about abuse is higher when compared to the study finding conducted by Sugg in 1999 [31]. Other presentations of domestic violence related to psychological, psychosomatic or antenatal problems which are more subtle to its relation to domestic violence were asked regarding abuse by the clinicians in a much lesser frequency. This finding is similar the study by Sugg [31]. Clinicians failing to identify and to offer abused women help despite repeated presentations to health care facilities may cause them further abuse when they are send home to the same abusive environment.

Most clinicians in this study reported lack of time as a barrier to ask for domestic violence. This finding is similar to that reported in Sugg et al [27]. Nearly a third of the clinicians were still unsure on how to ask regarding domestic violence among their patients which should raise major concern. It would seem reasonable to suggest that the clinicians have not been fully equipped on how to deal with domestic violence cases during their undergraduate or postgraduate training. The results also suggest the clinicians and the nursing staff received minimal or no training on violence management during their service.

Health care providers possess certain opinions and prejudices based on their own upbringing, culture and religious beliefs. These biases can affect their professional behavior including their intention to ask about abuse and create errors in clinical judgment in domestic violence cases. More than half of the clinicians and a third of the nursing staff reported a fear of offending patients in asking about domestic violence. This may be related to the underlying belief that domestic violence is a 'private matter' and not within the scope of medical treatment [29]. Nearly a third of clinicians and half of nurses endorsed the view that the abused person must have done something to trigger the abuse. This 'blaming the victim' attitude is a very negative way to address the person who has been victimized when the abuser should be the one to be blamed for using violence to resolve conflict [37].

Traditional beliefs regarding the family privacy, family unity and gender role was found to have posed difficulties to health care providers in their management of domestic violence [29]. However, many abused women do not mind being asked about violence and would like the health care providers to be more pro-active in asking questions on abuse [12,25,28,38]. Furthermore, health care providers need to be aware that domestic violence is indeed a major medical problem and they have important roles to play in its detection and management [39].

Having a safe environment will also enable the health care providers to identify domestic violence. There should be a place for the health care providers to have a private consultation with the victim without the presence of the abuser. In this study, a very small proportion of the participants expressed concern on their safety but a large proportion was concerned about the safety of their patients with in a violent environment.

Perceived self-efficacy plays an important role in the management of various medical conditions. Most clinicians in this study were more confident in asking about smoking and alcohol intake rather than asking about different kinds of abuse. Most of them perceived lack of self-efficacy in the overall management of domestic violence, including the use of strategies to help the abused person and a lack of access to different management information. All these negatively impact on the health care provider's ability to adequately care for abused person or abusers. Factors, such as inadequate training or the perception of poor success in management of these cases are relevant [30].

There is no mandatory reporting for domestic violence in Malaysia. However, in this study, a proportion of health care providers have indicated that they would still report cases of abuse to police despite abused women's refusal to give consent. Not respecting the patient's autonomy can be considered as unethical and may represent institutional victimization. There should be support for the abused patients no matter what their decision is at that point of time. This will increase their self-esteem and confidence level, aspects of their self-image that may have been severely undermined by repeated abused by their partner [22].

Within institutional settings, having enabling factors for the management of domestic violence will make the health care providers more inclined to manage these cases. Less than three-quarters of the participants in this study had access to a social worker, while 10% of the participants were unsure of the availability of social workers to help them manage domestic violence cases. Less than a third of the participants knew of any written protocol for the management of domestic violence. Not even half of the participants knew of the existence of non-governmental organization that can support management of domestic violence. The health care providers may feel inadequate in helping the abused victims with the lack of knowledge on the availability of various domestic violence resources.

## Conclusion

Primary health care providers in this study are more inclined to perceive domestic violence negatively and to manage it inappropriately with their limited skill and training in detection and management resulted from inadequate knowledge in domestic violence in general. Negative personal values also impact adversely on health care providers' detection and management.

### Implications from this study

Based on this study, primary health care providers need to receive training in domestic violence management and to have more information related to domestic violence in order to improve their management of domestic violence. The training should include theory of violence behavior as a way to change their views on violence victims and abuser; the proper management of domestic violence and ways to assist victims with safety plans. The primary health care providers need to be aware of local information related to domestic violence such as the prevalence, some legal aspect of it and the resources around them. The dearth of information regarding domestic violence management in Malaysia, particularly in primary health care setting demand more vigorous study. A detailed qualitative exploration within this area with a larger survey may provide a better understanding on specific issues brought up in this study.

## Competing interests

The authors declare that they have no competing interests.

## Authors' contributions

SO provided the initial concept of the study, conducted data collection, performed the statistical analysis and drafted the manuscript. NAMA participated in the design of the study and data collection. Both authors read and approved the final manuscript.
